# Ultrasound in Dual Nerve Impairment after Proximal Radial Nerve Lesion

**DOI:** 10.1371/journal.pone.0127456

**Published:** 2015-05-20

**Authors:** Alexandra B Lämmer, Stefan Schwab, Axel Schramm

**Affiliations:** Department of Neurology, Friedrich-Alexander-University Erlangen-Nuremberg, Erlangen, Germany; University of Palermo, ITALY

## Abstract

**Introduction:**

Sonography in classical nerve entrapment syndromes is an established and validated method. In contrast, few publications highlight lesions of the radial nerve, particularly of the posterior interosseus nerve (PIN).

**Method:**

Five patients with a radial nerve lesion were investigated by electromyography, nerve conduction velocity and ultrasound. Further normative values of 26 healthy subjects were evaluated.

**Results:**

Four patients presented a clinical and electrophysiological proximal axonal radial nerve lesion and one patient showed a typical posterior interosseous nerve syndrome (PINS). The patient with PINS presented an enlargement of the PIN anterior to the supinator muscle. However four patients with proximal lesions showed an unexpected significant enlargement of the PIN within the supinator muscle.

**Conclusion:**

High-resolution sonography is a feasible method to demonstrate the radial nerve including its distal branches. At least in axonal radial nerve lesions, sonography might reveal abnormalities far distant from a primary proximal lesion site clearly distinct from the appearance in classical PINS.

## Introduction

Diagnosis of peripheral nerve lesions, in particular nerve entrapment syndromes, is primarily based on clinical and electrophysiological findings. In the recent past, high- resolution sonography became an efficient method of visualising lesions of peripheral nerves in an inexpensive and time-saving way. Particularly nerve entrapment syndromes, such as carpal tunnel syndrome of the median nerve and cubital tunnel syndrome of the ulnar nerve, are well described and validated [1–4]. Various studies show a high correlation between electrophysiological findings, such as conduction velocity and distal motor latency, and cross-sectional area (CSA) in sonographic examination [5, 6].

In contrast, there are few reports of sonographic findings concerning the radial nerve and in particular the posterior interosseus nerve (PIN) [7–14]. Whereas proximal radial nerve lesions mostly involve triceps, brachioradialis and extensor carpi radialis muscle, posterior interosseous nerve syndrome (PINS) is characterised by weakness of the finger and ulnar wrist extension without sensory involvement. Pathogenesis of PINS includes typical local compression of the nerve by the Arcade of Frohse, as well as by ganglia, lipoma or abnormal vessels [8, 15, 12]. The sonographic appearance includes a typical swelling anterior to the radial tunnel [8, 14].

To evaluate the sonographic appearance of radial nerve lesions, we examined five patients by means of electromyography, nerve conduction velocity (NCV) and high- resolution ultrasound. Since available data are rare, we further assessed normative values of radial nerve sonography in 26 healthy volunteers.

## Materials and Methods

Five patients (one female, four male, mean age 44.0 ± 7.9 years, range 30–68 years) with a clinical lesion of the radial nerve and 26 controls (mean age 46.92 ± 18,35 years, range 22–86 years) were investigated. Details of the clinical data of all patients are shown in Table 1. The research was conducted in accordance with the Declaration of Helsinki and was approved by the ethic committee of the Friedrich-Alexander University of Erlangen-Nuremberg. Patients had given written consent.

**Table 1:** Patient data.

	Age	Duration of symptoms [Month]	MRC [x/5]	Sensory loss	SCV	Electromyography	APD [mm] right/left APD_path/healthy_
**Patients with proximal lesion**							
**1**	30	3 month	T 5 WFE 1–2 radial deviation	Yes	No SNAP	No myography	1.2/0.7 1.71
**2**	29	3 weeks	T 4 BR 0 WFE 1	Yes	No SNAP	T: AD +++, single potentials BR: AD +, no potentials at activity EDC: AD +, normal activity	1.2/0.8 1.5
**3**	68	3 month	T 2 WFE 0	Yes	No SNAP	T: AD +++, neurogen pattern EDC: AD +++, normal activity	1.4/0.5 2.8
**4**	48	6 month	T 2 WFE 4 radial deviation	No	reduced SNAP	T, EDC: normal BR: AD +++, neurogen pattern ECU: no AD, neurogen pattern	1.4/0.8 1.75
**Patient with classical PINS**							
**5**	43	3 years	T 5 WFE 3 radial deviation	No	normal SNAP	ECR normal EDC AD ++, neurogen pattern	1.3/1.1 1.18

Detailed clinical, electrodiagnostic and sonographic findings of all patients. (AD—active denervation, APD—anterior-posterior diameter, BR—brachioradialis muscle, ECR—extensor carpi radialis muscle, ECU—extensor carpi ulnaris muscle, EDC—extensor digitorum communis muscle, MRC—medical researche council, SNAP—sensory nerve action potential, SCV—sensory conduction velocity, T—triceps brachii muscle, WFE—wrist/finger extension, + mild, ++ moderate, +++ severe)

### Clinical and electrophysiological examination

Patients underwent standardised neurological and electrophysiological examination including nerve conduction velocity (NCV) study and electromyography. NCV study consisted of sensory conduction velocity (SCV), and sensory nerve action potential (SNAP) amplitude of the superficial radial nerve. After stimulation at the forearm, SNAP was recorded at the thumb with ring electrodes. SCV (m/s) of the radial nerve was calculated and SNAP amplitudes were determined peak to peak (μV). Standard needle electromyography was performed according to clinical presentation, mainly including triceps muscle, brachioradialis muscle and extensor digitorum communis muscle, to assess spontaneous electrical activity in the resting muscle, and configuration and recruiting pattern of motor unit potentials with voluntary contraction.

### High-resolution ultrasound

All patients and volunteers were investigated using a high-resolution ultrasound system with an 18 MHz linear array transducer (Acuson S2000, Siemens Medical Systems, Erlangen, Germany). The patient was seated on a chair with arms resting in a flexed position, the hands in pronation. We performed longitudinal and transverse scans of the proximal radial nerve and the posterior interosseous nerve.

In the healthy volunteers, the cross-sectional area (CSA) of the radial nerve was measured: a) at the spiral groove, b) at the level of the elbow before splitting and c) the PIN before entering the supinator muscle. The antero-posterior diameter (APD) of the PIN was measured within the supinator muscle.

In the patients, the radial nerve was scanned from the spiral groove to the forearm to search for focal abnormalities. CSA and APD measurements were performed at the inner border of the thin hyperechogenic rim delimiting the nerve. APD ratio of lesion side versus healthy side (APD_path/healthy_) was calculated.

### Statistical Analysis

APD were presented as means ± SD and the statistical differences were tested by the nonparametric Mann-Whitney U-Test. The level of statistical significance was set at *p* < 0.05.

## Results

### Clinical and electrophysiological examination

All patients showed typical radial nerve palsy, with at least paresis of finger extension and wrist extension (with radial deviation in three cases, Table 1). Four out of five patients displayed a proximal lesion with paresis of the triceps or brachioradialis muscle, three out of five patients showed sensory deficits. Electrophysiology confirmed a proximal lesion site in these cases and demonstrated axonal injury due to absent or reduced SNAP. Electromyography showed signs of active denervation in the triceps or brachioradialis muscle in three cases.

One patient showed typical signs of a posterior interosseous nerve lesion, with normal SNAP amplitude and pathological electromyography of the extensor digitorum communis, but not of the extensor carpi radialis muscle.

The controls showed completely normal neurological examination.

### High-resolution ultrasound

**Fig 1:**
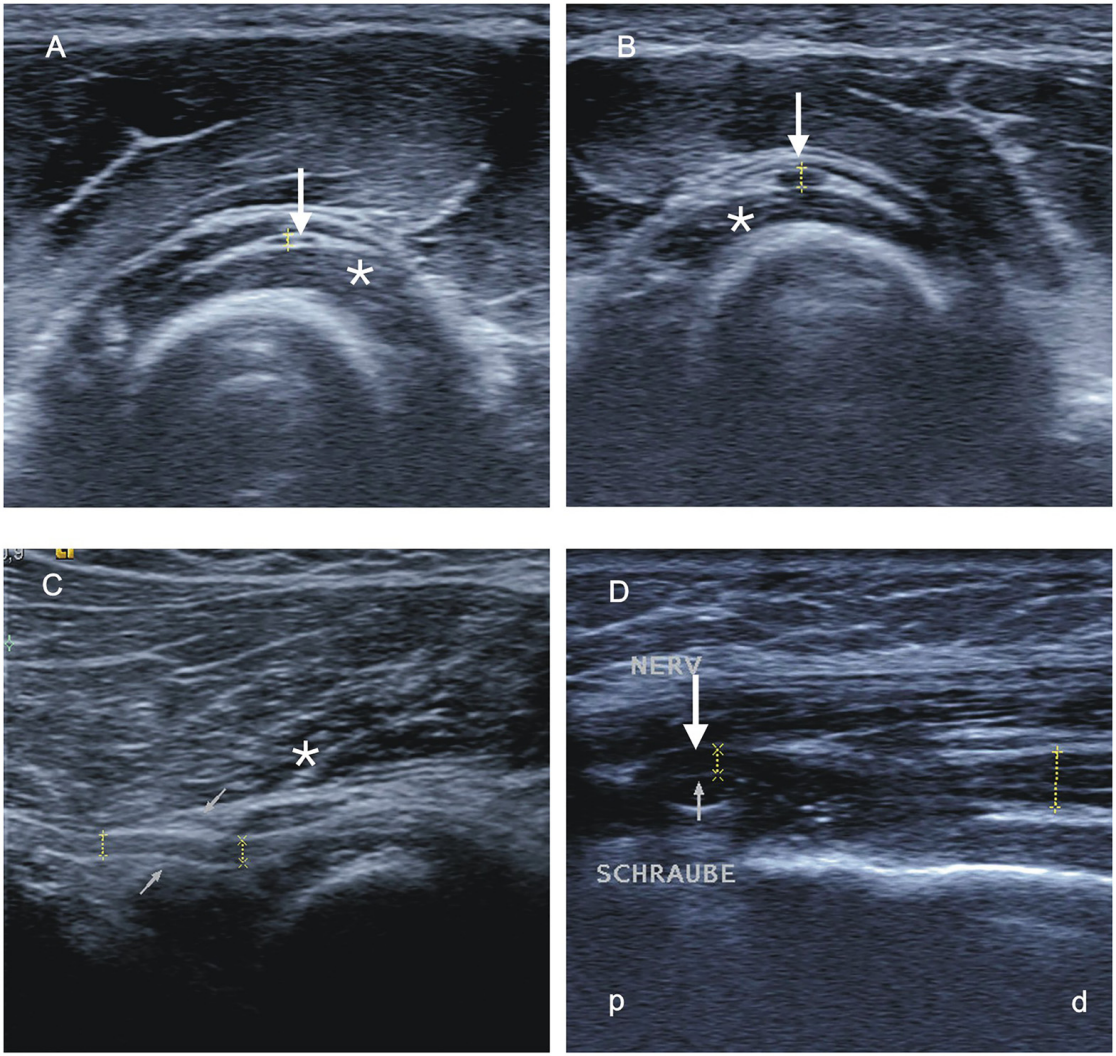
Sonographic studies of a patient with a proximal radial nerve lesion. (A, B) Transversal study of the posterior interosseous nerve (PIN; arrow) within the supinator muscle (asterisk) on the healthy side (A) in comparison to the affected side (B) with a significant swelling of the PIN within the muscle. (C) Longitudinal study of the PIN with a swelling before entering the Arcade of Frohse (thin arrows) and within the supinator muscle (asterisk). (D) Longitudinal study of the radial nerve in the distal upper arm with impression of the nerve (arrow) by a screw (thin arrow). p—proximal, d—distal

**Fig 2:**
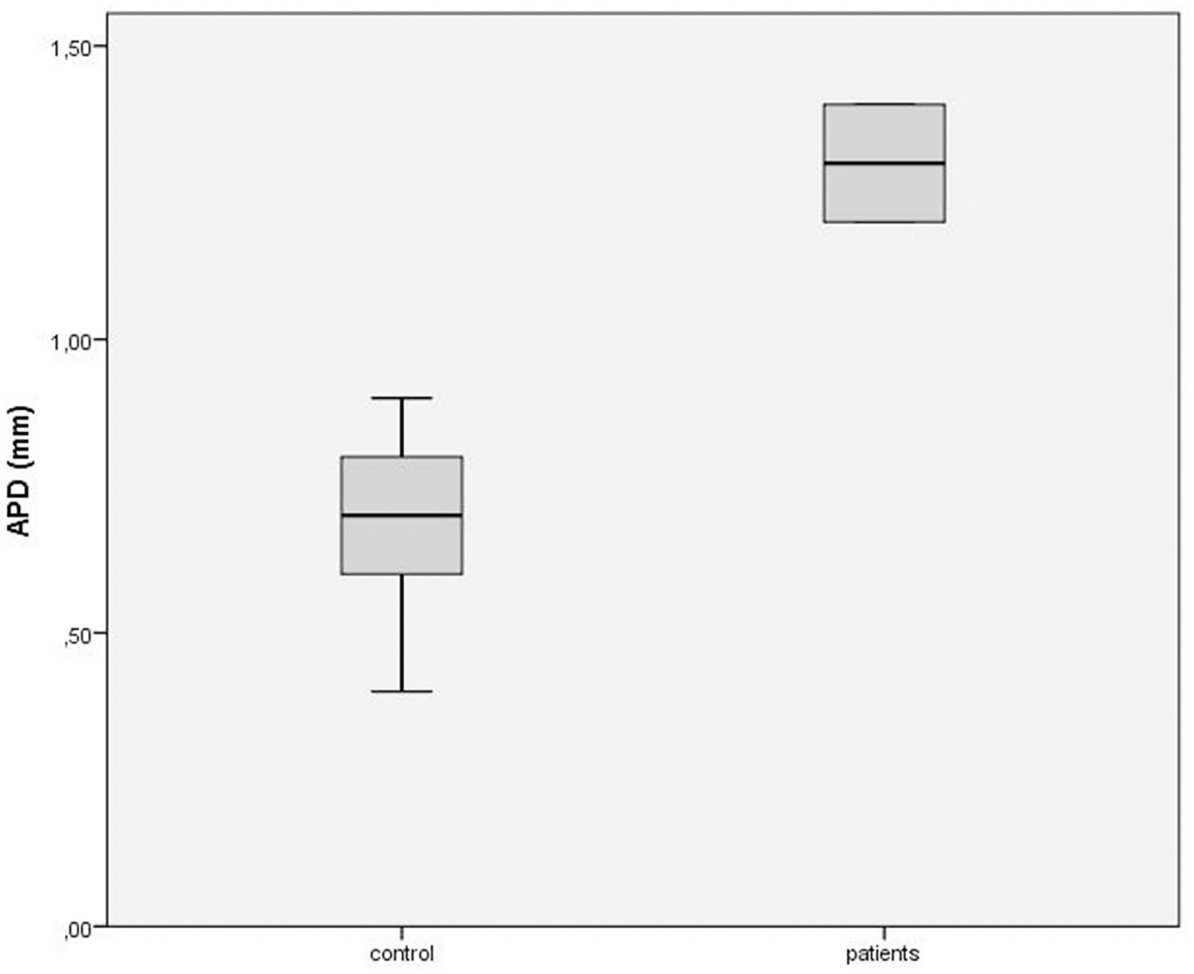
Antero-posterior diameter (APD) of posterior interosseous nerve (PIN). Antero-posterior diameter (APD) of PIN in four patients shows a significant swelling of the PIN in comparison to controls. ** *p*<0.001

All four patients with a proximal radial nerve lesion showed a significant swelling of the PIN within the supinator muscle (Fig 1B, 1C) in comparison to the healthy side (Fig 1A; APD_path_ 1.30 ± 0.1 mm versus APD_healthy_ 0.67 ± 0.13, *p*<0.001, APD_path/healthy_ = 1.96 ± 0.81) and in comparison to controls (APD_path_ 1.30 ± 0.1 mm versus APD_norm_ 0.67 mm ± 0.18 mm, *p*<0.001; Fig 2). In one patient with proximal radial nerve lesion, a corresponding deterioration by an osteosynthesis screw could be detected by sonography (Fig 1D).

In the healthy controls, no side-to-side difference was observed (APD_right_ 0.65 ± 0.15 versus APD_left_ 0.69 ± 0.1, n.s.; Table 2).

**Table 2:** Normative values.

	Spiral grove [CSA; mm^2^] n = 44	Before splitting at elbow [CSA; mm^2^] n = 52	PIN anterior to supinator muscle [CSA; mm^2^] n = 50	PIN within supinator muscle [APD; mm] n = 50	APD _right/left_ [mm] n = 23
**Mean**	0.59 ± 0.19	0.54 ± 0.14	0.21 ± 0.01	0.68 ± 0.12	0.94 ± 0.17

Cross sectional area (CSA) and antero-posterior diameter (APD) of the radial nerve and the posterior interosseus nerve (PIN) of 26 healthy volunteers (= 52 nerves) presented as mean ± standard deviation.

**Fig 3:**
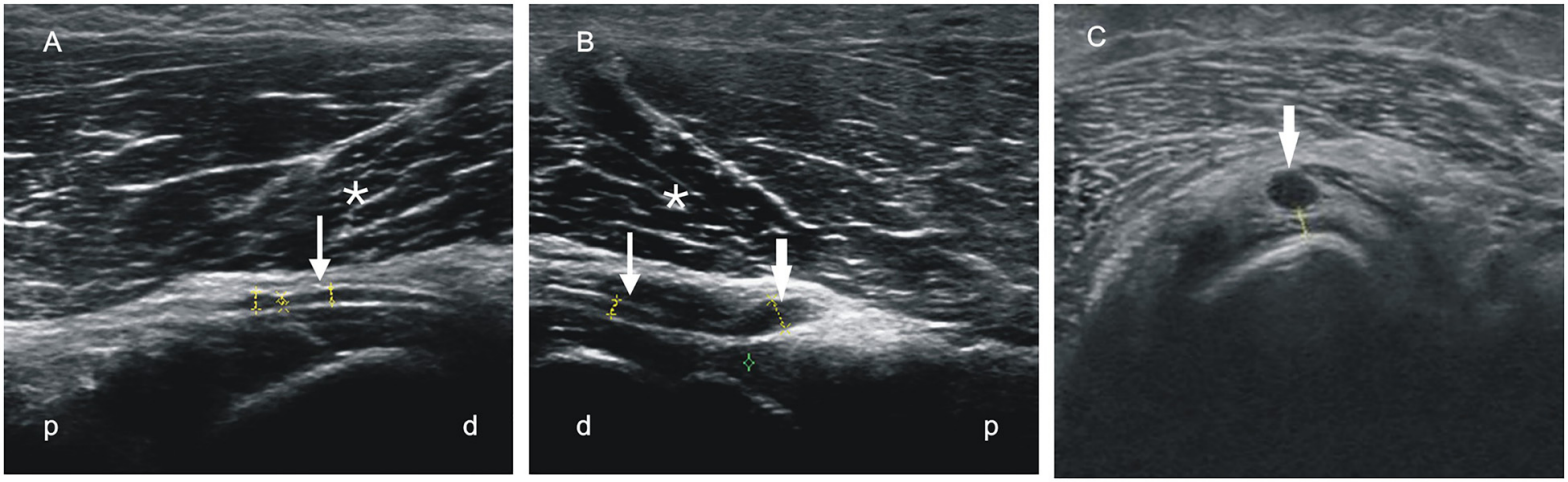
Sonographic studies of the patient with a posterior interosseous nerve (PIN) syndrome. (A, B) Longitudinal study of healthy PIN (A) and the affected side (B) with significant swelling (thick arrow) before entering the supinator muscle (asterisk). (C) Transversal study with a significant swelling of the PIN (arrow) anterior to the supinator muscle. p—proximal, d—distal

The patient with PINS showed a focal swelling of the nerve before entering the supinator muscle on the affected side (Fig 3B, 3C), but not within the supinator muscle itself or on the healthy side (Fig 3A).

## Discussion

Our experience and presented data confirm former studies reporting that the radial nerve could be easily visualised with high-resolution ultrasound, from the proximal upper arm [7, 10, 15] in its entirety to its distal branches, such as the PIN [8, 9, 11–14]. We could demonstrate sonomorphological, focal lesions corresponding to clinical examination and/or neurophysiology in two patients: one with an affection of the radial nerve through an osteosynthesis screw in the upper arm, as well as one with a classical PINS, showing focal swelling of the PIN at the Arcade of Frohse before entering the supinator muscle but not inside the muscle.

Interestingly, all four patients with proximal radial nerve palsy showed an unexpected swelling of the PIN anterior to and especially within the supinator muscle, distant from the assumed proximal lesion site.

For quantification of nerve swelling, especially in the case of the flat partially bean- like shape of the median or ulnar nerve, the CSA should be consulted. By contrast, quantification of the PIN is usually performed by the antero-posterior diameter (APD), which is suspected to correlate with the CSA, since the PIN shows mostly a round morphology [8]. When reviewing the present literature, normative values of the PIN are described once in 10 volunteers (APD_mean_ 1.31 mm, range 1.0–1.5mm) [14].

In comparison, our own normative data show smaller values which might be due to methodical differences. The authors did not describe the exact method and localization of APD measurement. As we measured at the inner border of the thin hyperechogenic rim delimiting the nerve, our normative values could be lower and a comparison of both values might not be valid.

In the present study, one patient showed a typical PINS with enlargement of the nerve before entering the supinator muscle, which has been previously described [8, 13, 14]. In general, sonographic abnormalities in nerve entrapment syndromes are well characterized, including changes in nerve shape with abrupt flattening of the nerve at the compression site and hypoechoic swelling proximal to it. Changes of echotexture consist of rarefied or completely lost fascicular structure, caused primarily by intraneuronal venous congestion and oedema [16–18]. Nevertheless, there might be signs of inflammatory hyperaemia with intra- and perineuronal flow signals on colour scan [18]. In one patient with a proximal lesion, we detected the focal entrapment with swelling anterior to a narrowing osteosynthesis screw. In the remaining cases with proximal lesions, we could not demonstrate a focal lesion even though clinical and electrophysiological presentation proved a proximal lesion of the radial nerve. However routine scans of the radial nerve were performed from the mid part of the humerus at the level of the spiral groove and then followed down to the elbow. It is assumed that the lesion sites were more proximal, especially since the patients show an involvement of the triceps muscle, whose branches arise in the proximal part of the upper arm.

All presented cases of proximal paresis show an unclear distal swelling of the posterior interosseous nerve within the supinator muscle far distal from the proximal lesion site. Swelling of the nerve distal to the compression site is rare and has been primarily described only twice as an atypical double nerve lesion after humeral fracture. Both cases demonstrate a focal enlargement of the radial or median nerve at the proximal lesion site, as well as at an additional distal site [7, 19]. In the case concerning the radial nerve [7], the authors describe a swelling at the inlet of the Arcade of Frohse, but figures seem to show the PIN within the supinator muscle, but not at the inlet. So this finding might be in accordance with our own data. The authors hypothesized an indirect mechanism of stretching due to the fracture while the PIN is fixed at the inlet of the Arcade of Frohse.

Since three out of four of our patients suffered from traumatic nerve injury but all presented an axonal lesion, an alternative hypothesis might be possible: in an experimental axonal sciatic lesion model, Bendszus *et al*. [20] could demonstrate an increased nerve signal in magnetic resonance imaging and nerve oedema on histology at the entire nerve distal to the lesion site. Axonal degeneration in our patients could therefore have induced oedema, which might be accentuated in a closed muscular compartment such as the supinator muscle. Nevertheless, the pathogenesis of sonomorphological changes distal from a primary lesion site or in double nerve lesions is not yet resolved. To verify our observations studies in larger cohorts including other nerves, pure demyelinating lesions and follow-up examinations might be of special interest.

## Conclusion

High-resolution sonography is a valid method of demonstrating the radial nerve and posterior interosseous nerve, particularly in entrapment syndromes. At least in axonal radial nerve lesions, sonography might reveal abnormalities distant from a proximal primary lesion site clearly distinct from the appearance in classical PINS. Nevertheless the consistency, the course and the significance for prognosis of these findings have to be clarified by future studies.

## References

[1] Klauser AS, Halpern EJ, De Zordo T, Feuchtner GM, Arora R, Gruber J et al Carpal tunnel syndrome assessment with US: value of additional cross-sectional area measurements of the median nerve in patients versus healthy volunteers. Radiology 2009; 250:171–177. 10.1148/radiol.2501080397 19037017

[2] Kutlay M, Colak A, Simsek H, Ozturk E, Senol MG, Topuz K et al Use of ultrasonography in ulnar nerve entrapment surgery—a prospective study. Neurosurg Rev 2009; 32:225–232. 10.1007/s10143-008-0162-4 18797947

[3] Wiesler ER, Chloros GD, Cartwright MS, Shin HW, Walker FO. Ultrasound in the diagnosis of ulnar neuropathy at the cubital tunnel. J Hand Surg Am 2006; 31:1088–1093. 16945708 10.1016/j.jhsa.2006.06.007

[4] Duncan RE. Median nerve compression with tumescent fluid administration. Plast Reconstr Surg 1999; 103:1095 10077123

[5] Volpe A, Rossato G, Bottanelli M, Marchetta A, Caramaschi P, Bambara LM et al Ultrasound evaluation of ulnar neuropathy at the elbow: correlation with electrophysiological studies. Rheumatology (Oxford) 2009; 48:1098–1101. 10.1093/rheumatology/kep167 19567661

[6] Mondelli M, Filippou G, Gallo A, Frediani B. Diagnostic utility of ultrasonography versus nerve conduction studies in mild carpal tunnel syndrome. Arthritis Rheum 2008; 59:357–366. 10.1002/art.23317 18311762

[7] Liotta G, Granata G, Librante A, di Pasquale A, Caliandro P, Martinoli C et al Atypical double nerve lesion after humeral fracture: diagnosis by ultrasound. Muscle Nerve 2011; 41:287–288. 10.1002/mus.21580 19918776

[8] Kinni V, Craig J, van Holsbeeck M, Ditmars D. Entrapment of the posterior interosseous nerve at the arcade of Frohse with sonographic, magnetic resonance imaging, and intraoperative confirmation. J Ultrasound Med 2009; 28:807–812. 19470822 10.7863/jum.2009.28.6.807

[9] Joy V, Therimadasamy A, Cheun CY, Wilder-Smith E. Diagnostic utility of ultrasound in posterior interosseous nerve syndrome. Arch Neurol 2009; 66:902–903. 10.1001/archneurol.2009.109 19597095

[10] Toros T, Karabay N, Ozaksar K, Sugun TS, Kayalar M, Bal E. Evaluation of peripheral nerves of the upper limb with ultrasonography: a comparison of ultrasonographic examination and the intra-operative findings. J Bone Joint Surg Br 2009; 91:762–765. 10.1302/0301-620X.91B6.22284 19483229

[11] Visser LH. High-resolution sonography of the superficial radial nerve with two case reports. Muscle Nerve 2009; 39:392–395. 10.1002/mus.21246 19208405

[12] Yamazaki H, Kato H, Hata Y, Murakami N, Saitoh S. The two locations of ganglions causing radial nerve palsy. J Hand Surg Eur Vol 2007; 32:341–345. 17331627 10.1016/J.JHSB.2006.09.014

[13] Chien AJ, Jamadar DA, Jacobson JA, Hayes CW, Louis DS. Sonography and MR imaging of posterior interosseous nerve syndrome with surgical correlation. AJR Am J Roentgenol 2003; 181:219–221. 12818863 10.2214/ajr.181.1.1810219

[14] Bodner G, Harpf C, Gardetto A, Kovacs P, Gruber H, Peer S et al Ultrasonography of the accessory nerve: normal and pathologic findings in cadavers and patients with iatrogenic accessory nerve palsy. J Ultrasound Med 2002; 21:1159–1163. 12369671 10.7863/jum.2002.21.10.1159

[15] Jou IM, Wang HN, Wang PH, Yong IS, Su WR. Compression of the radial nerve at the elbow by a ganglion: two case reports. J Med Case Reports 2009; 3:7258 10.4076/1752-1947-3-7258 19830153 PMC2726493

[16] Bianchi S. Ultrasound of the peripheral nerves. Joint Bone Spine 2008; 75:643–649. 10.1016/j.jbspin.2008.07.002 18823807

[17] Martinoli C, Schenone A, Bianchi S, Mandich P, Caponetto C, Abbruzzese M et al Sonography of the median nerve in Charcot-Marie-Tooth disease. AJR Am J Roentgenol 2002; 178:1553–1556. 12034637 10.2214/ajr.178.6.1781553

[18] Martinoli C, Bianchi S, Gandolfo N, Valle M, Simonetti S, Derchi LE. US of nerve entrapments in osteofibrous tunnels of the upper and lower limbs. Radiographics 2000; 20 Spec No:S199-213; discussion S213-197. 10.1148/radiographics.20.suppl_1.g00oc08s199 11046171

[19] Liotta G, di Pasquale A, Lucchetta M, Alberti M, Padua L. Ultrasound view of a traumatic two level median nerve lesion. Muscle Nerve 2011; 43(5): 767–8. 10.1002/mus.22011 21404301

[20] Bendszus M, Wessig C, Solymosi L, Reiners K, Koltzenburg M. MRI of peripheral nerve degeneration and regeneration: correlation with electrophysiology and histology. Exp Neurol 2004; 188:171–177. 15191813 10.1016/j.expneurol.2004.03.025

